# Meeting of the Southern Section of the American Laryngological, Rhinological and Otological Society, in Atlanta, Ga., March 28

**Published:** 1898-03

**Authors:** Dunbar Roy

**Affiliations:** Secretary


### MEETING OF THE SOUTHERN SECTION OF THE AMERICAN LARYNGOLOGICAL, RHINOLOGICAL AND OTOLOGICAL SOCIETY, IN ATLANTA, GA., MARCH 28.—PRELIMINARY PROGRAM.

1. Address by the Chairman. Dr. A. W. Calhoun, Atlanta, Ga.
2. Labyrinthine Vertigo. Dr. John Hey Williams, Asheville, N. C.
3. Chromatic Audition. Dr. J. L. Minor, Memphis, Tenn.
4. Report of a Case of Hemorrhage after the Removal of Adenoid Vegetations. Dr. Ross P. Cox, Rome, Ga.
5. Hypertrophy of the Lingual Tonsil, its Symptoms and its Treatment. Dr. D. A. Kuyk, Richmond, Va.
6. Thrombosis of the Lateral Sinus, Dependent upon Suppurative Otitis Media. Dr. E. B. Dench, New York.
7. Othematoma and Pericondrotis of the Auricle. Dr. John O. McReynolds, Dallas, Texas.
8. Tracheotomy for Foreign Bodies in the Air Passages; Report of Twenty-seven Successful Cases. Dr. Willis F. Westmoreland, Atlanta, Ga.

9. Empyema of the Accessory Nasal Cavities. Dr. Ruffin A. Wright, Mobile, Ala.

10. The Influence on Development of the Nervous System of the Child by Adenoid Vegetations in the Nasopharynx. Dr. E. P. Sale, Memphis, Tenn.

11. Nasal Fibromata with Report of Cases. Dr. L. M. Crichton, Atlanta, Ga.

12. A Form of Primary Nasal Diphtheria. Dr. E. C. Ellett, Memphis, Tenn.

13. The Importance of Examining the Nose in Troublesome Coughs. Dr. Alfred C. Palmer, Richmond, Va.

14. Intubation of the Larynx for Membranous Stenosis. Dr. Bernard Wolff, Atlanta, Ga.

15. Mouth Breathing. Dr. Norburne B. Jenkins, Knoxville, Tenn.

16. Mental Disturbance in Turbinate Hypertrophies or Nasal Stenosis. Dr. John F. Woodward, Norfolk, Va.

17. A Discovery in the Physiology of the Ear. Dr. W. F. Cole, Waco, Texas.

18. Middle Ear Catarrh; Some Original Deductions. Dr. Maury M. Stapler, Macon, Ga.

19. Ethmoiditis and Report of Case. Dr. B. F. Travis, Chattanooga, Tenn.

20. Mastoid Inflammation, with a Report of Two Interesting Cases. Dr. T. E. Mitchell, Columbus, Ga.

21. Mouth Breathing in Children, Particularly as a Result of Adenoids. Dr. Arthur G. Hobbs, Atlanta, Ga.

22. The Nasopharynx in Laryngeal Diseases. Dr. N. C. Steel, Chattanooga, Tenn.

23. Cholesteatoma of the Mastoid Antrum. Dr. S. L. Ledbetter, Birmingham, Ala.

24. Diseases of the Accessory Sinuses. Dr. Alex. W. Stirling, Atlanta, Ga.

25. Clinical Miscellanies from Private Practice. Dr. Frank M. Mullins, Ft. Worth, Texas.

26. Empyema of the Accessory Sinuses of the Nose. Dr. Frank M. Hanger, Staunton, Va.

27. A Good Hemostatic after Nasal Operations. Dr. L. B. Grandy, Atlanta, Ga.

28. Hypertrophic Rhinitis and its Treatment. Dr. J. F. Hill, Memphis, Tenn.

29. The Serum Treatment of Ozena. Dr. W. E. Campbell, Atlanta, Ga.

30. Peritonsillar Abscess. Dr. Dunbar Roy, Atlanta, Ga.

All the hotels have given reduced rates and a large meeting is expected. The meeting will be called to order at 10 A.M. in the ballroom of the Kimball House. The profession is cordially invited.

DUNBAR ROY,
Secretary.

---

THE ATLANTA MEDICAL AND SURGICAL JOURNAL is one of the oldest medical journals in the South and as such should be in the office of every Georgia physician. Our State is known everywhere for its progressiveness and for the fact that Georgians are proud of their own State. Then let the physicians stand by their home journal by writing articles for its pages, by subscribing and getting their friends to subscribe. We want medical items from all over the State and invite our friends to send us such. Our pages will be filled with *practical medicine* and abstracts of subjects which will interest the busy practitioner. Two dollars a year is a small amount for the pleasure and information which you will get from the pages of the JOURNAL.

Publish your communications and medical writings in your home journal, where they will be read by your professional friends who know you. They will be much more appreciated.

This number is the beginning of our fifteenth volume (new series). We shall endeavor to make it and succeeding volumes more and more worthy of indorsement and support on the part of our many readers.

---

THE American Medical Association meets at Denver, June 7th to 10th. One of the features of the gathering will be an excursion from Denver to Salt Lake City and return, via the Denver and Rio Grande, Colorado Midland, and Rio Grande Western Railways, through the "Heart of the Rockies," furnishing a splendid opportunity to view the most magnificent scenery on the American Continent. Salt Lake City is an ideal summer resort, and the bathing at Saltair in the Great Salt Lake—inland salt sea nearly a mile above sea level—is superb in June. There are more attractions in and about Salt Lake City than any place in the world. Later notice will appear in this publication giving rates for this excursion and all details. In the meantime send to F. A. Wadlaigh, G. P. A., Rio Grande Western Railway, Salt Lake City, for copy of pamphlets on Salt Lake City and the Rocky Mountains.

---

ONE OF THE JOURNAL'S oldest subscribers is Dr. J. J. Devine, of Tom Bean, Texas. In a late letter, renewing his subscription, the doctor reminds us that he has been taking THE JOURNAL for twenty-four years. We desire to thank Dr. D. for his continued interest and loyalty, which we trust will be uninterrupted for another twenty-four years.

